# Roll-call data and ideological estimates from two consecutive constitutional bodies: The Chilean Constitutional Convention (2021–2022) and the Constitutional Council (2023)

**DOI:** 10.1016/j.dib.2026.113113

**Published:** 2026-07-30

**Authors:** Juan Rozas, Pablo A. Henríquez, Aldo Mascareño

**Affiliations:** a Centro de Estudios Públicos (CEP), Monseñor Sótero Sanz 162, Providencia, 7500011, Santiago, Chile; b Millennium Nucleus for Social Data Science (SODAS), Santiago, Chile; c Facultad de Administración y Economía, Universidad Diego Portales, Av. Santa Clara 797, Huechuraba, 8580000, Santiago, Chile

**Keywords:** Ideal point estimation, Legislative behavior, Plenary voting, W-NOMINATE, Bayesian IRT, Dynamic scaling, Political polarization

## Abstract

We provide roll-call vote records, member-level metadata, and ideological position estimates for the 205 members of the two consecutive constitutional bodies elected in Chile between 2021 and 2023: the Constitutional Convention (July 2021–July 2022, n = 155) and the Constitutional Council (June–November 2023, n = 50). The two bodies produced 5919 plenary roll-call votes (4738 in the Convention and 1181 in the Council), covering all proposals, articles, and amendments voted on the floor of each assembly. We excluded procedural votes, votes on non-binding agreements, and votes on internal organizational matters.

We estimated ideological positions independently within each body using three methods: the W-NOMINATE model; a Bayesian item-response theory model that produces posterior means and 95% credible intervals; and a dynamic model that tracks within-body ideological movement across four temporal periods.

The two bodies were elected under distinct institutional rules: the Convention applied gender parity, seventeen reserved seats for indigenous peoples, and electoral rules that facilitated independent candidacies; the Council operated under party or coalition lists, with a single supernumerary indigenous seat allocated subject to a minimum vote threshold. The proposals drafted by each body were rejected in national plebiscites in September 2022 and December 2023. Member-level variables include political party, electoral pact, district or constituency, region, date of birth, sex, reserved-seat status, and a political-sector classification (Left, Center-Left, Center, Center-Right, or Right).

These data can support comparative research on constitutional processes and analyses in political science, public policy, and institutional studies.

Specifications Table

**Table:** 

Subject	Social Sciences
Specific subject area	Roll-call votes and ideal-point estimates for the Chilean Constitutional Convention (2021–2022) and Constitutional Council (2023).
Type of data	Table (.csv format) Figure (.pdf format) Processed data Supporting materials (R scripts to reproduce the ideological estimates and figures; README file)
Data collection	We collected roll-call votes from the JSON web services of the Plenario Virtual platforms of the Constitutional Convention (https://sala.cconstituyente.cl) and the Constitutional Council (https://www.procesoconstitucional.cl/salaconsejo). We curated member-level metadata from public records of the National Library of the Chilean Congress (BCN). We processed and cleaned the data using R (v4.3.0). We estimated ideological positions using three methods: W-NOMINATE, Bayesian IRT, and dynamic IRT.
Data source location	Institution: Convención Constitucional and Consejo Constitucional de Chile. City/Region: Santiago, Metropolitan Region. Country: Chile. Latitude and longitude: 33°26′18″ S, 70°39′12″ W. Type of data: Secondary data (roll-call votes published by both bodies). Primary data sources: Official roll-call platforms of the Constitutional Convention (https://www.chileconvencion.cl) and the Constitutional Council (https://www.procesoconstitucional.cl).
Data accessibility	Repository name: Harvard Dataverse Data identification number: 10.7910/DVN/CE7WHF Direct URL to data: https://doi.org/10.7910/DVN/CE7WHF
Related research article	None.

## 1 Value of the Data

• These data provide the first systematic record of roll-call votes and ideological estimates for two consecutive constitution-drafting bodies in Chile, supporting research on legislative behavior outside standard legislative cycles.
• The two bodies were elected under contrasting institutional rules (gender parity, reserved indigenous seats, and independent candidacies in the Convention; closed party lists in the Council), allowing researchers to assess how electoral design shapes ideological composition and bloc cohesion.
• Coverage of all plenary roll-call votes (5919 votes across 205 members) supports analyses of polarization, cohesion, and fragmentation, as well as methodological work on ideal-point estimation in small-*N*, high-*J* settings.
• Estimates from three methods (W-NOMINATE, Bayesian IRT, and dynamic IRT) allow comparison of frequentist and Bayesian approaches, analysis of within-body ideological trajectories, and robustness checks across estimation strategies.
• The files can be linked to electoral, plebiscite, and party-system data sources for research on representation and coalition behavior.

## 2 Background

Between October 2019 and December 2023, Chile carried out a constitution-drafting cycle involving two consecutive elected bodies and three national plebiscites. A social uprising in October 2019 led to a cross-party agreement that opened a constitutional pathway, ratified in an entry plebiscite in October 2020. The Constitutional Convention (July 2021–July 2022) comprised 155 members elected with gender parity, seventeen reserved seats for indigenous peoples, and rules that allowed independent candidates to compete on equal terms with party-affiliated candidates. Its proposal was rejected by 61.89 percent of voters in September 2022. The Constitutional Council (June–November 2023) comprised 50 members elected through party or coalition lists under the senatorial electoral system; its proposal was rejected by 55.76 percent of voters in December 2023.

Prior studies have produced ideological estimates and roll-call analyses of these processes [[1], [2], [3]], and several initiatives have released estimates or voting data through public repositories and analytical platforms [[4], [5], [6], [7]]. None of these provides an integrated database of both bodies. The dataset documented in this article [8] compiles the complete plenary roll-call record of both bodies, harmonizes member-level metadata, and provides ideological estimates under three methods in an integrated and systematically documented structure, with scripts to reproduce the estimation pipeline.

## 3 Data Description

We organized the dataset [8] into three subfolders within the data repository, comprising twelve CSV files. The first subfolder (matrix/) contains the roll-call vote matrices for each body (member × vote). The second subfolder (metadata/) contains member-level and vote-level metadata. The third subfolder (ideological estimation/) contains the ideological position estimates, distributed in six body-specific files (one per body and estimation method), mirroring the body-specific organization of the roll-call matrices and metadata files. The repository also includes the R scripts required to reproduce the ideological estimates and the figures from the distributed files. Table 1 reports the structure and dimensions of each file.

**Table 1:** Files included in the repository.

File	Content	Rows	Columns
*Roll-call vote matrices*			
matrix_convention_2021_22.csv	Convention votes (member × vote)	155	4743
matrix_council_2023.csv	Council votes (member × vote)	50	1186
*Member-level metadata*			
members_convention.csv	Convention members	155	16
members_council.csv	Council members	50	16
*Vote-level metadata*			
votes_convention.csv	Convention votes (one row per vote)	4738	11
votes_council.csv	Council votes (one row per vote)	1181	11
*Ideological estimates*			
ideology_wnominate_convention.csv	W-NOMINATE static estimates, Convention	154	7
ideology_wnominate_council.csv	W-NOMINATE static estimates, Council	50	7
ideology_bayesian_convention.csv	Bayesian IRT static estimates with 95% CI, Convention	154	11
ideology_bayesian_council.csv	Bayesian IRT static estimates with 95% CI, Council	50	11
ideology_dynamic_convention.csv	Dynamic IRT estimates (long format; 4 rows per member), Convention	616	9
ideology_dynamic_council.csv	Dynamic IRT estimates (long format; 4 rows per member), Council	200	9

All files use UTF-8 encoding and follow a consistent naming convention. Each identifier combines a body prefix (CONV for the Convention, CONS for the Council), an entity code (M for member, V for vote), and a zero-padded sequential index. Member identifiers therefore run from CONV_M001 to CONV_M155 in the Convention and from CONS_M001 to CONS_M050 in the Council. Vote identifiers run from CONV_V00001 to CONV_V04738 and from CONS_V00001 to CONS_V01181, ordered chronologically within each body.

### 3.1 Roll-call vote matrices

Two files contain the universe of plenary roll-call votes, one per body: matrix_convention_2021_22.csv and matrix_council_2023.csv. Each row corresponds to one member; the first five columns provide identifiers, and each subsequent column corresponds to one roll-call vote. Vote columns are sorted chronologically in ascending order, from CONV_V00001 to CONV_V04738 in the Convention and from CONS_V00001 to CONS_V01181 in the Council. Table 2 describes the column structure of the roll-call matrices. Table 3 reports the distribution of vote outcomes in each body.

**Table 2:** Column structure of the roll-call vote matrices.

Column	Description
n	Sequential row index for internal reference
member_id	Unique member identifier (CONV_M001–CONV_M155 or CONS_M001–CONS_M050)
first_name	First name(s) of the member
paternal_surname	Paternal surname
maternal_surname	Maternal surname
CONV_V##### or CONS_V#####	One column per roll-call vote, ordered chronologically. Cell values are Yea, Nay, Abstention, or NA when the member was not in office on the day of the vote or the vote was not registered.

**Table 3:** Distribution of vote outcomes across both bodies.

Votes	Convention Cells	Convention %	Council Cells	Council %
Yea	392,999	53.5	45,504	77.1
Nay	232,526	31.7	7959	13.5
Abstention	68,483	9.3	5334	9.0
NA	40,382	5.5	253	0.4
**Total**	**734,390**	**100.0**	**59,050**	**100.0**

The original source files use Spanish vocabulary (“AFAVOR”, “ENCONTRA”, “ABSTENCION”). We recoded these values to the standardized English vocabulary documented in Table 2 during the curation process, preserving a one-to-one correspondence between the original and recoded values.

The contrast in the number of NA cells between the two bodies reflects the different durations of each process. The Council operated over five months with near-universal attendance, producing 253 missing values (0.4% of total). The Convention operated over twelve months, producing 40,382 missing values (5.5% of total), which include routine absences and the resignation of one member (Rodrigo Rojas Vade, March 2022).

### 3.2 Member-level metadata

Two files describe the members of each body, members_convention.csv (155 rows, 16 columns) and members_council.csv (50 rows, 16 columns), with one row per member. Table 4 describes the columns, and Table 5 reports the composition of both bodies by political sector.

**Table 4:** Column structure of the member-level metadata files. Both files share an identical column structure (16 columns each).

Column	Description
member_id	Unique member identifier (CONV_M001–CONV_M155 or CONS_M001–CONS_M050)
original_id	Original member identifier from the source website
first_name	First name(s) of the member
paternal_surname	Paternal surname
maternal_surname	Maternal surname
official_name	Full official name as registered by the body
sex	Sex (Male or Female)
date_of_birth	Date of birth (ISO format)
age_at_start	Age in years at the start of the body’s tenure
political_party	Political party at the time of election
electoral_pact	Electoral pact or list
district	Electoral district (Convention) or constituency (Council)
region	Region of Chile (Indigenous Peoples for reserved seats)
reserved_seat	Whether the seat was reserved for indigenous peoples
body	Body to which the member belongs (Convention or Council)
political_sector	Political sector (Left, Center-Left, Center, Center-Right, or Right)

**Table 5:** Composition of both bodies by political sector.

Body	Political sector	Members
Convention	Left	76
	Center-Left	27
	Center	15
	Right	37
Council	Left	11
	Center-Left	6
	Center-Right	5
	Right	28
**Total**		**205**

The Convention comprised 155 members elected in May 2021: 138 across 28 electoral districts and 17 in seats reserved for indigenous peoples. Fifty-one members were elected to the Council in May 2023: 50 across 16 constituencies, one per region, plus a single supernumerary seat reserved for indigenous peoples. One elected member did not take office, so the Council operated with 50 members. The political_sector variable classifies each member into one of five sectors (Left, Center-Left, Center, Center-Right, or Right).

### 3.3 Vote-level metadata

Two files describe the individual votes in each body: votes_convention.csv (4738 rows) and votes_council.csv (1181 rows), with one row per roll-call vote. Each row records the date, session number, subject matter, and outcome of the vote, together with its aggregated vote totals. Both files share the same column structure, described in Table 6.

**Table 6:** Column structure of the vote-level metadata files. Both files share an identical column structure (11 columns each).

Column	Description
vote_id	Unique vote identifier (CONV_V00001–CONV_V04738 or CONS_V00001–CONS_V01181). Ordered chronologically.
original_vote_id	Original vote identifier from the source website
session_number	Session number within the body
date	Date and time of the vote (POSIXct, UTC). The Convention records date only (time set to 00:00:00); the Council records full timestamps with hours, minutes, and seconds.
subject	Subject matter of the vote (free text, in Spanish)
outcome	Overall outcome of the vote (Approved or Rejected)
total_yea	Number of Yea votes
total_nay	Number of Nay votes
total_abstention	Number of Abstention votes
total_no_vote	Number of members who did not cast a vote. In the Council, this value is reported directly by the source. In the Convention, it is derived as n_active(t) − (yea + nay + abstention), where n_active(t) = 155 for votes before March 15, 2022, and 154 from that date onward, reflecting the resignation of Rodrigo Rojas Vade accepted by TRICEL.
body	Body to which the vote belongs (Convention or Council)

The Convention held 91 plenary sessions between July 13, 2021, and June 28, 2022. The Council held 29 plenary sessions between August 24 and October 30, 2023.

### 3.4 Ideological estimates

We distribute the ideological position estimates in six files, one per body and scaling method, mirroring the body-specific structure of the remaining data components and keeping each file focused on a single estimation approach. The two files of each method share an identical column structure; across methods, the files share a common set of identifier columns and differ in the estimation-specific columns.

We provide estimates for 204 of the 205 members. We exclude Rodrigo Rojas Vade (CONV_M047) from the estimates files because he cast only 163 votes before ceasing to participate in the Convention; his official resignation was accepted by the Electoral Tribunal (TRICEL, https://tribunalcalificador.cl/) on March 15, 2022. We retain his roll-call record up to that point in the matrix file.

The three estimation methods are:

• **W-NOMINATE** [9], a frequentist scaling method implemented in the wnominate R package. It provides two-dimensional static estimates (wnom_D1, wnom_D2) within each body.
• **Bayesian item-response theory** [10], implemented through the ideal() function of the pscl package. It provides two-dimensional posterior means (bay_D1, bay_D2) with 95% credible intervals.
• **Dynamic item-response theory** [11], implemented through the dynIRT() function of the emIRT package. It provides one-dimensional estimates (dyn_D1) within each body across four temporal periods, with 95% confidence intervals derived from the posterior variances.

Tables 7, 8, and 9 document the columns of each file.

**Table 7:** Column structure of the W-NOMINATE estimate files, ideology_wnominate_convention.csv (154 rows) and ideology_wnominate_council.csv (50 rows). Both files share an identical column structure (7 columns each), with one row per member.

Column	Description
body	Body to which the member belongs (Convention or Council)
member_id	Unique member identifier
first_name	First name(s)
paternal_surname	Paternal surname
maternal_surname	Maternal surname
wnom_D1	W-NOMINATE point estimate on the first dimension
wnom_D2	W-NOMINATE point estimate on the second dimension

**Table 8:** Column structure of the Bayesian IRT estimate files, ideology_bayesian_convention.csv (154 rows) and ideology_bayesian_council.csv (50 rows). Both files share an identical column structure (11 columns each), with one row per member.

Column	Description
body	Body to which the member belongs (Convention or Council)
member_id	Unique member identifier
first_name	First name(s)
paternal_surname	Paternal surname
maternal_surname	Maternal surname
bay_D1	Bayesian posterior mean on the first dimension
bay_D1_low	Lower bound of the 95% credible interval (D1)
bay_D1_high	Upper bound of the 95% credible interval (D1)
bay_D2	Bayesian posterior mean on the second dimension
bay_D2_low	Lower bound of the 95% credible interval (D2)
bay_D2_high	Upper bound of the 95% credible interval (D2)

**Table 9:** Column structure of the dynamic IRT estimate files, ideology_dynamic_convention.csv (616 rows) and ideology_dynamic_council.csv (200 rows). Both files share an identical column structure (9 columns each) and are in long format, with one row per member-period combination, yielding four rows per member across the four temporal periods.

Column	Description
body	Body to which the member belongs (Convention or Council)
member_id	Unique member identifier
first_name	First name(s)
paternal_surname	Paternal surname
maternal_surname	Maternal surname
period_num	Temporal period index (0 to 3). Periods are equal-sized quartile partitions of the chronologically ordered roll-call sequence within each body, not substantive stages of the constitutional process
dyn_D1	Dynamic IRT point estimate of the first-dimension position in the period
dyn_D1_low	Lower bound of the 95% confidence interval (derived from the posterior variance)
dyn_D1_high	Upper bound of the 95% confidence interval (derived from the posterior variance)

**Fig. 1:**
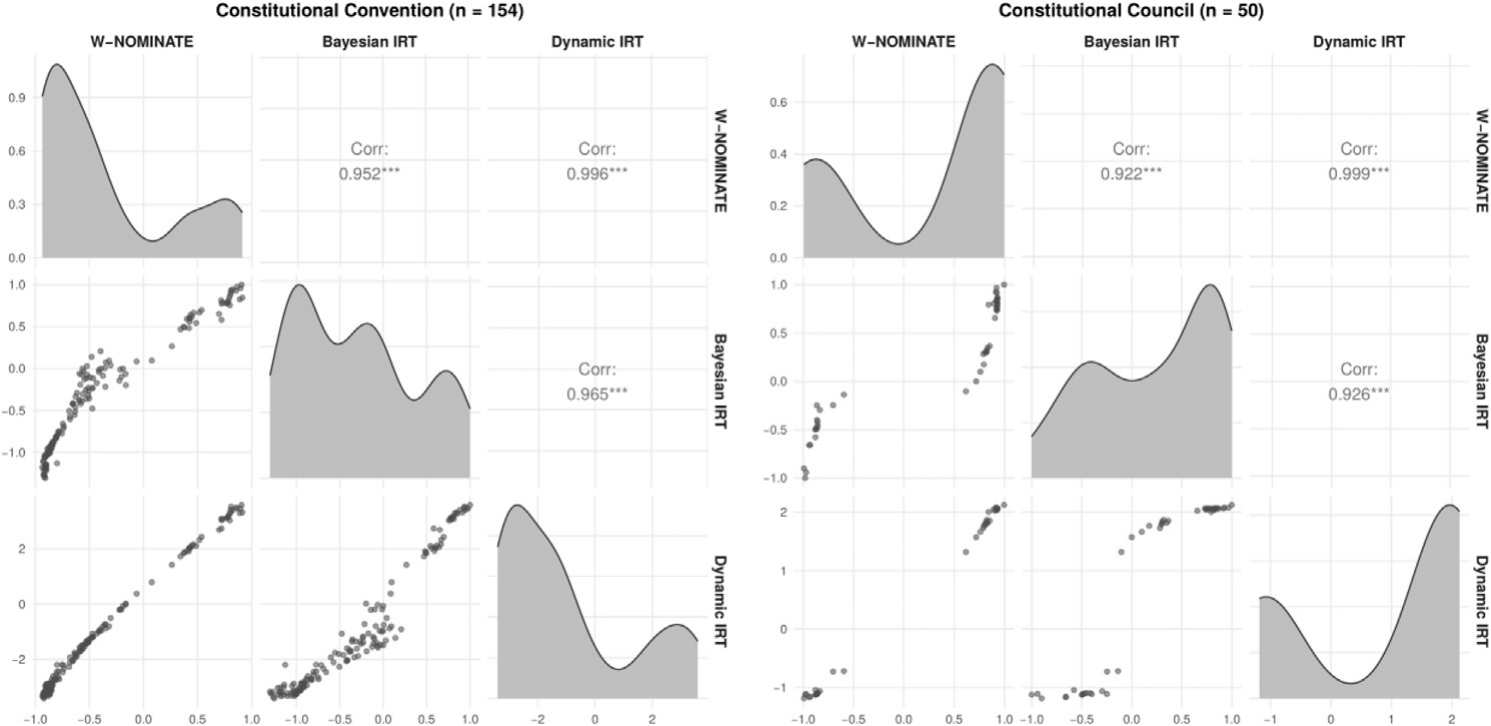
Pairwise correlation of D1 ideological estimates across methods, separately for the Constitutional Convention (left) and the Constitutional Council (right). Dynamic IRT estimates are averaged across the four temporal periods. Diagonal panels show the marginal density of each estimate; lower-triangle panels display pairwise scatterplots; upper-triangle panels report Pearson correlation coefficients.

**Fig. 2:**
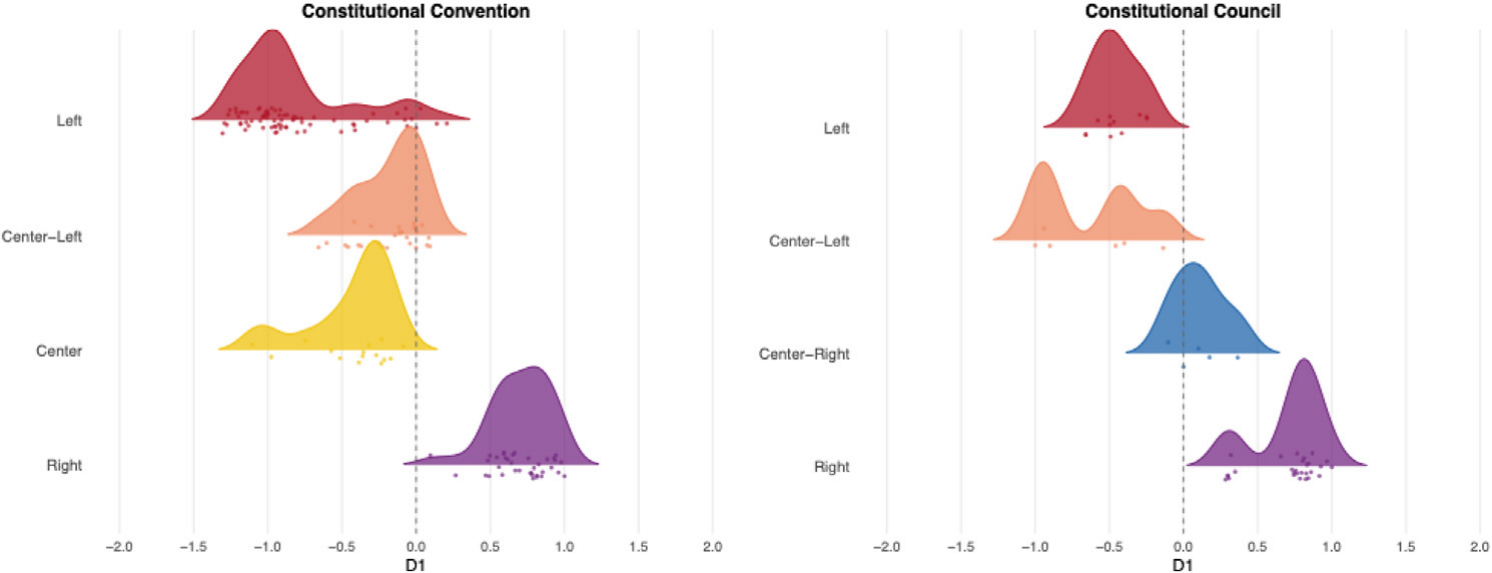
Distribution of Bayesian D1 ideological estimates by political sector, shown in two separate panels with body-specific horizontal axes: Constitutional Convention (left) and Constitutional Council (right). Within each panel, political sectors are arranged from Left (top) to Right (bottom).

**Fig. 3:**
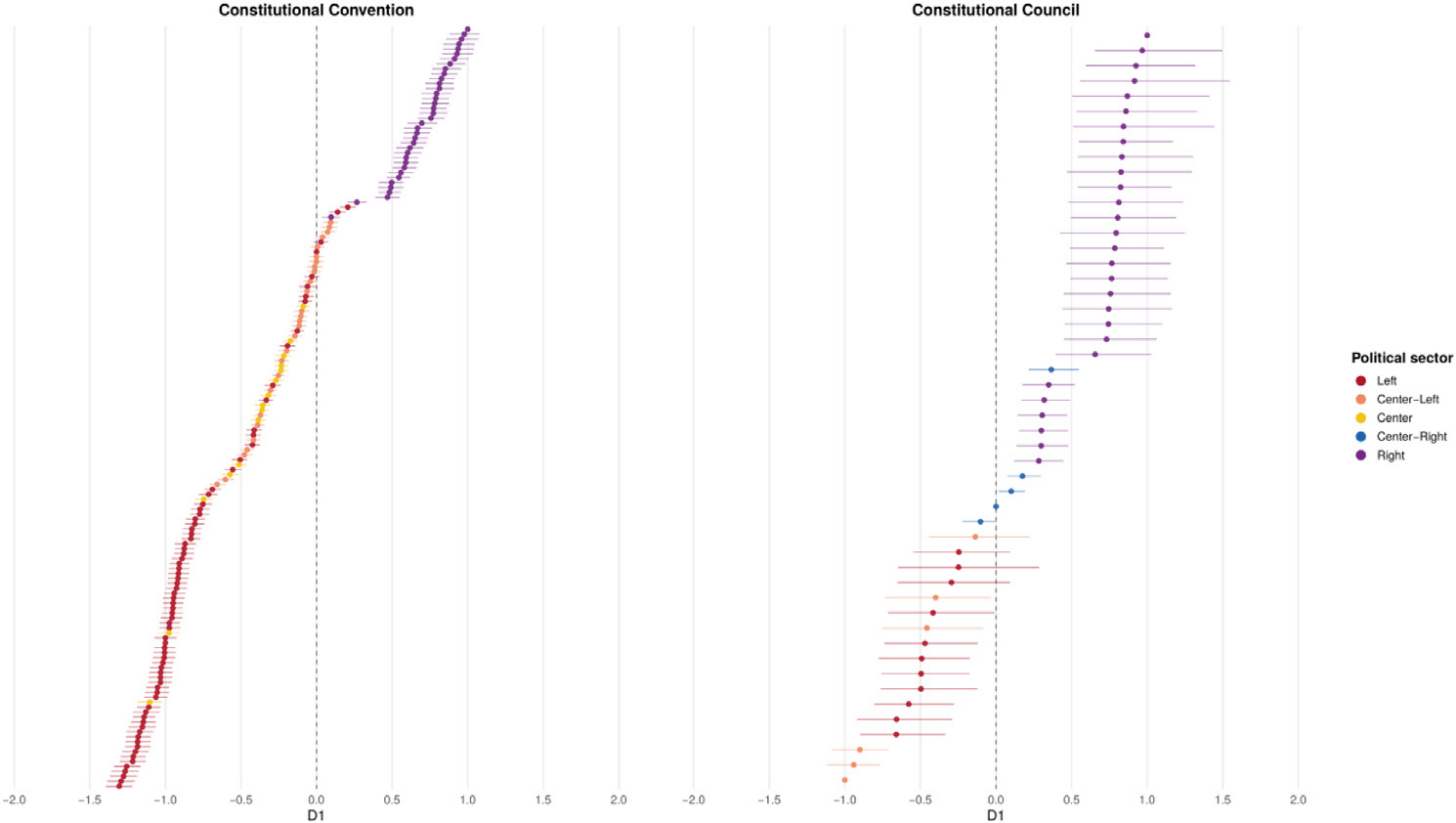
Bayesian D1 ideal-point estimates with 95% credible intervals, ordered by point estimate within each body and colored by political sector.

Three figures complement the tables above. Fig. 1 shows the pairwise correlation of D1 estimates across methods. Fig. 2 shows the distribution of Bayesian D1 estimates by political sector within each body. Fig. 3 shows the Bayesian D1 estimates of all members with their 95% credible intervals.

## 4 Experimental Design, Materials and Methods

### 4.1 Data sources and acquisition

We obtained roll-call data from the JSON web services that powered the *Plenario Virtual* interfaces of the two constitutional bodies. The Constitutional Convention published vote-level data through the service endpoints under https://sala.cconstituyente.cl, and the Constitutional Council published equivalent data under https://www.procesoconstitucional.cl/salaconsejo.

Using the httr, jsonlite, purrr, and dplyr packages, we implemented a data collection routine that queried four nested endpoints: (i) the list of plenary sessions of each body; (ii) session-level metadata for each session, including the date, session number, and start and end times; (iii) the agenda items of each session; and (iv) the individual roll-call votes recorded for each item. For each vote, the service returned the list of members in attendance, their individual choice (“AFAVOR”, “ENCONTRA”, “ABSTENCION”), and the aggregate totals. We recoded these values into the standardized vocabulary used across both bodies in the distributed matrices: Yea, Nay, and Abstention.

### 4.2 Member-level metadata

We manually curated member-level metadata from public records published by the National Library of the Chilean Congress (Biblioteca del Congreso Nacional, BCN). The BCN maintains a section dedicated to the constitutional process that includes biographical profiles for all 155 members of the Constitutional Convention (https://www.bcn.cl/historiapolitica/convencionales_constituyentes/index.html) and all 50 members of the Constitutional Council (https://www.bcn.cl/historiapolitica/proceso_constitucional/consejo_constitucional.html). Each profile reports the member’s full name, date of birth, sex, electoral district or constituency, political party at the time of election, electoral pact, and whether the seat was reserved for indigenous peoples.

From these public records, we assembled the variables in members_convention.csv and members_council.csv, cross-checked them against the records of the Electoral Service of Chile (https://www.servel.cl), and aligned them to the member identifiers used in the roll-call matrices through a name-matching procedure.

### 4.3 Ideological estimation

We derived ideal-point estimates from three roll-call scaling methods, fit independently to each body. We carried out estimation in R 4.3.0 using the wnominate, pscl, and emIRT packages, and set a common random seed (set.seed(123)) before each estimation call to ensure reproducibility.

**W-NOMINATE.** We computed two-dimensional static estimates through the wnominate() function [9]. We used minvotes = 20 (minimum number of votes per legislator to be included), lop = 0.025 (minimum proportion of the minority side for a vote to be retained), and trials = 3 (number of random starting configurations). We set the polarity of the two dimensions through the polarity argument, using Rocío Cantuarias (D1) and Beatriz Sánchez (D2) in the Convention, and Antonio Barchiesi (D1) and Lorena Gallardo (D2) in the Council. We report the estimates in wnom_D1 and wnom_D2.

**Bayesian item-response theory.** We computed two-dimensional Bayesian estimates through the ideal() function of the pscl package [10]. We used 25,000 MCMC iterations, a burn-in of 5000, and thinning of 25, yielding 800 retained samples per legislator per dimension. The function uses standard normal priors on ideal points and diffuse priors on item parameters. We identified the latent space by post-processing the posterior samples (postProcess()) with an affine transformation that fixes three members per body at known positions: Cristina Dorador at (−1, 0), Rocío Cantuarias at (1, 0), and Beatriz Sánchez at (0, 1) in the Convention; Miguel Littín at (−1, 0), Antonio Barchiesi at (1, 0), and Lorena Gallardo at (0, 1) in the Council. These anchors, chosen for their publicly recognized ideological profiles, orient D1 consistently with the W-NOMINATE solution; by construction, the six anchored members have degenerate (zero-width) credible intervals. We report posterior means as point estimates in bay_D1 and bay_D2, and 95% credible intervals from the posterior quantiles in the corresponding _low and _high columns.

**Dynamic item-response theory.** We computed one-dimensional dynamic estimates through the dynIRT() function of the emIRT package [11]. We divided the chronologically ordered roll-call sequence of each body into four equal-sized segments defined by the quartiles of the distribution of vote dates, indexed t = 0, 1, 2, 3, so that each period contains approximately one-quarter of the roll calls held in that body. These periods are analytical partitions of the roll-call sequence rather than substantively differentiated stages of each constitutional process: plenary voting was concentrated in specific sittings and episodes of intensive deliberation, so a period boundary may fall within a single session or deliberative episode. Users interested in substantively defined periodizations can construct alternative partitions from the vote dates and session numbers recorded in votes_convention.csv and votes_council.csv. The model links legislator ideal points across these four periods through a random-walk prior. We specified standard normal priors on starting positions (x ∼ N(0, 1)), zero-mean priors on item parameters with identity covariance, and an evolution variance of ω² = 0.01. We controlled convergence with a tolerance of 10⁻⁶ and a maximum of 500 EM iterations. The within-period likelihood is invariant to a joint sign reversal of member positions and item discrimination parameters, so neutral or random starting values can lead the EM algorithm to a locally optimal solution in which one period is reflected relative to the others. To avoid this, we initialized each member’s position in all periods at their static W-NOMINATE first-dimension estimate. We report the point estimates (dyn_D1) together with the lower and upper bounds of the 95% confidence interval derived from the posterior variance (dyn_D1_low, dyn_D1_high). We record the period index in the period_num column.

**Exclusion criterion.** One member of the Convention (Rodrigo Rojas Vade, identifier CONV_M047) cast only 163 roll-call votes before ceasing to participate, and his official resignation was accepted by TRICEL on March 15, 2022. Although this exceeds the W-NOMINATE minvotes threshold of 20, the number of votes is too small for stable estimation in the dynamic model and makes cross-period comparison unreliable. We exclude this member from all three methods in the distributed estimates files; we retain his roll-call record up to the date of effective withdrawal in the matrix file. We therefore provide estimates for 154 of the 155 Convention members and all 50 Council members (204 in total).

**Model fit diagnostics.** W-NOMINATE provides standard goodness-of-fit measures based on classification accuracy. For the Convention, the one-dimensional solution achieved an aggregate proportional reduction in error (APRE) of 0.668 and a correct classification rate of 91.5%; the two-dimensional solution improved fit to APRE = 0.707 and 92.5% correct classification. For the Council, the one-dimensional solution achieved APRE = 0.961 and 98.9% correct classification; the two-dimensional solution did not improve fit further.

## Limitations

The ideological estimates are not directly comparable across the two bodies. The Constitutional Convention (2021–2022) and the Constitutional Council (2023) were separate constituent bodies with non-overlapping membership and distinct institutional rules, deliberation lengths, and voting agendas, so their estimates do not lie on a common scale.

The ideological estimates cover 204 of the 205 members. One Convention member (CONV_M047) cast too few votes for stable estimation and appears only in the matrix file, as detailed in the Experimental Design, Materials and Methods section. This affects analyses that characterize the full 155-member composition of the Convention from the ideological estimates.

The distribution of vote outcomes in the Council is highly skewed, with 77.1% of recorded votes affirmative against 53.5% in the Convention. Ideal-point estimators perform best when voting is balanced, so the high classification accuracy observed for the Council (98.9%) partly reflects this limited variation in outcomes.

Finally, the four temporal periods of the dynamic estimates are equal-sized partitions of each body’s chronologically ordered roll-call sequence; they do not correspond to substantive stages of the constitutional processes, and period boundaries may fall within a single session or deliberative episode.

## Ethics Statement

The authors have read and follow the ethical requirements for publication in Data in Brief and confirm that the current work does not involve human subjects, animal experiments, or any data collected from social media platforms.

## CRediT Author Statement

**Juan Rozas:** Conceptualization, Methodology, Software, Data curation, Formal analysis, Visualization, Writing – original draft. **Pablo A. Henríquez:** Methodology, Software, Validation, Writing – review & editing. **Aldo Mascareño:** Conceptualization, Supervision, Funding acquisition, Writing – review & editing.

## Data Availability

DataverseRoll-Call Voting Data and Ideological Estimates from the Chilean Constitutional Processes (2021–2023) (Original data).
